# Recent Advances in Antiviral Therapy for Chronic Hepatitis C

**DOI:** 10.1155/2016/6841628

**Published:** 2016-01-31

**Authors:** Akihiro Tamori, Masaru Enomoto, Norifumi Kawada

**Affiliations:** Department of Hepatology, Osaka City University Graduate School of Medicine, 1-4-3 Asahimachi, Abeno-ku, Osaka 545-8585, Japan

## Abstract

Hepatitis C virus (HCV) infection is a major worldwide health problem. Chronic infection induces continuous inflammation in the liver, progression of hepatic fibrosis, eventual cirrhosis, and possible hepatocellular carcinoma. Eradication of the virus is one of the most important treatment aims. A number of promising new direct-acting antivirals (DAAs) have been developed over the past 10 years. Due to their increased efficacy, safety, and tolerability, interferon-free oral therapies with DAAs have been approved for patients with HCV, including those with cirrhosis. This review introduces the characteristics and results of recent clinical trials of several DAAs: NS3/4A protease inhibitors, NS5A inhibitors, and NS5B inhibitors. DAA treatment failure and prognosis after DAA therapy are also discussed.

## 1. Introduction

Chronic hepatitis C (CHC) due to infection with hepatitis C virus (HCV) affects approximately 170 million people worldwide and is the most common cause of chronic liver disease [1]. Of HCV-infected individuals, 20% to 30% eventually develop liver cirrhosis or hepatocellular carcinoma (HCC). The primary aims of anti-HCV therapy for patients with CHC are prevention of progression to cirrhosis and development of HCC. A combination of pegylated interferon (PEG-IFN) and ribavirin (RBV) completely eradicates HCV in up to 40% to 50% of treatment-naïve patients with high viral loads of HCV genotype 1b [2, 3]. Elongation of the treatment period or retreatment improves the rate of sustained virological response (SVR) in some patients with CHC [4–7]. In patients achieving SVR, IFN-based therapy has improved hepatic fibrosis and prevented the development of HCC. However, only limited numbers of patients show beneficial antiviral effects of IFN-based therapy. The effect depends on the patient's genetic background, presence of hepatic fibrosis, age, HIV coinfection, and other factors. In addition, IFN-based therapy has some adverse effects that may lead to poor drug adherence or treatment discontinuation.

**Figure 1:**
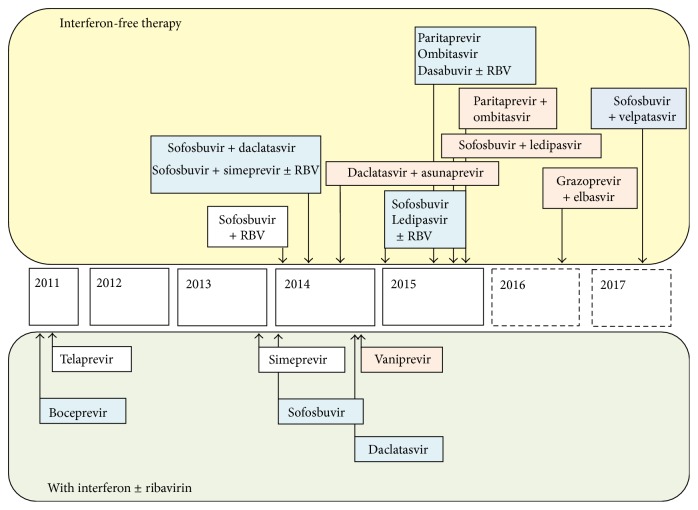
Treatment of HCV with DAAs that are approved or are going to be approved.

Recently, direct-acting antiviral (DAA) regimens were approved for anti-HCV therapy and have been evaluated. The first-generation protease inhibitors telaprevir (TVR) and boceprevir (BOC) were approved as DAA combination therapy with PEG-IFN and RBV [8–11]. Although triple therapy achieves a higher SVR rate than does conventional IFN-based therapy, treatment is associated with severe adverse effects. Neither the American Association for the Study of Liver Diseases (AASLD) Practice Guidelines nor the European Association for the Study of the Liver (EASL) Clinical Practice Guidelines for CHC recommend TVR or BOC triple therapy [12, 13]. Due to the development of new DAAs with better safety and stronger antiviral effects, it is expected that almost all patients with HCV infection will be able to achieve SVR in the near future (Figure 1). Therefore, it is necessary to consider the long-term prognosis of patients with CHC after eradication of HCV.

Here, we review recent developments in DAA therapy and discuss the management of patients with SVR.

## 2. Characteristics of DAAs

The development of an* in vitro* culture system for HCV has facilitated the search for agents with anti-HCV effects, and many such agents have undergone clinical trials for use as DAAs [14, 15]. DAAs are classified into three groups: NS3/4A serine protease inhibitors, NS5A inhibitors, and NS5B polymerase inhibitors (Tables 1 [Table tab2]–3). NS3/4A serine protease is required for self-cleavage during HCV replication, the NS5A region plays an important role in viral replication and assembly, and the NS5B region encodes RNA polymerase, which is necessary for HCV replication.

NS3/4A serine protease inhibitors consist of linear first-wave inhibitors and macrocyclic second-wave inhibitors. The NS5B polymerase inhibitors include nucleos(/t)ide types and nonnucleos(/t)ide types [16].

*In vitro* analysis showed that the antiviral effects of DAAs are dependent on the HCV genotype. In addition, some variants of HCV showed a high EC_50_ for each DAA. Some resistance-associated variants (RAVs) were detected in patients during the natural course of the disease. HCV variants with R155K or A156T in the NS3/4A protease show high resistance to the linear type inhibitors TVR and BOC. The resistance profiles to simeprevir (SMV), a macrocyclic inhibitor of NS3/4A protease, showed overlap with those to TVR and BOC. However, there are specific mutations that confer resistance to SMV [17]. RAV with Q80K was the most commonly observed variant at baseline in particular patients with genotype 1a. D168 mutation is a rarely observed variant associated with virological failure in patients with genotype 1 treated with SMV [18]. In addition, other macrocyclic protease inhibitors, including asunaprevir (ASV) [19], danoprevir, vaniprevir, and paritaprevir (ABT-450), show resistance profiles similar to those of SMV. RAVs with combinations of the mutations in NS3/4A have greater resistance to protease inhibitors than do single mutants. Fortunately, it is rare for such RAVs to emerge at baseline. The second-generation protease inhibitors grazoprevir (MK-5172) and sovaprevir (ACH-1625) [20, 21] have pan-genotypic activities that reduce viral escape through common resistance-associated mutations, such as R155K or Q80K variants.

The NS5A inhibitor daclatasvir (DCV) is a genotype-specific DAA that shows an extremely high antiviral effect against HCV genotype 1, particularly genotype 1b, compared with genotype 1a. In one study, variant viruses with L31M and/or Y93H, which are resistant to DCV, were detected in 4.2% and 14.5% of 214 DAA-naïve patients with HCV genotype 1b, respectively [22]. In another study, ledipasvir (LDV, GS-5885), ombitasvir (ABT-267), and elbasvir (MK-8742), all of which are also NS5A inhibitors, showed resistance profiles identical to that of DCV [23]. ACH-3102 and velpatasvir (GS-5816) are second-generation HCV NS5A inhibitors with potent broad genotype antiviral coverage and broad coverage of first-generation NS5A inhibitor RAVs.

Finally, compounds that inhibit NS5B are classified into two subclasses: nucleos(/t)ide competitive polymerase inhibitors and allosteric inhibitors of RNA polymerase (nonnucleos(/t)ide polymerase inhibitors; NNPIs). NNPIs have a high barrier to resistance [24] and appear to be effective across a broad range of viral genotypes. NNPIs induce conformational changes in the NS5B polymerase enzyme by binding to its various allosteric sites. These agents have a lower barrier of resistance and appear to be genotype specific. As a result of differences in sites of action on the polymerase, these two inhibitors have different mechanisms and potencies [16].

Sofosbuvir (SOF) is a uridine nucleotide prodrug NS5B inhibitor [25]. Following absorption, SOF is metabolized in hepatocytes, where it is converted to the active nucleoside triphosphate form. No dose adjustment of SOF is warranted in cases of mild, moderate, or severe hepatic fibrosis, although viral suppression may be slower among patients with Child-Pugh Class B and C liver disease. The safety and efficacy of SOF have not been established in patients with renal impairment (estimated glomerular filtration rate < 30 mL/min) or end-stage renal disease, including patients requiring hemodialysis [26]. S282T is a cross-resistance mutation within the NS5B polymerase to nucleos(/t)ide polymerase inhibitors, including SOF. However, this RAV is extremely rare in patients with a natural clinical course.

The structure of the NS5B polymerase resembles a characteristic “right hand motif” comprising finger, palm, and thumb domains, and at least five different allosteric binding sites at the thumb (sites 1 and 2) and palm (sites 3, 4, and 5) have been identified as targets for NNPIs. The NNPIs beclabuvir (BMS-791325), dasabuvir (ABT-333), and GS-9669 bind to sites 1, 3, and 2, respectively. Compared with NPIs, NNPIs have limitations in their antiviral effectiveness.

## 3. IFN-Based Therapy with DAAs

### 3.1. Genotypes 1/4

In clinical trials, addition of first-generation NS3/4A protease inhibitors, such as TVR [9, 10] or BOC [27, 28], substantially increased the rate of SVR to PEG-IFN and RBV. However, treatment is sometimes accompanied by severe adverse events, such as rash, pruritus, and anemia with TVR treatment and anemia and dysgeusia with BOC treatment, and use of first-generation protease inhibitors is currently not recommended.

Second-generation macrocyclic NS3/4A protease inhibitors, such SMV and vaniprevir, are generally well tolerated. The phase III QUEST-1/2 trials showed that SMV plus PEG-IFN and RBV for 12 weeks followed by PEG-IFN and RBV for 12 weeks or 36 weeks according to criteria for response-guided therapy resulted in SVR in 80% to 81% of treatment-naïve patients with genotype 1 infection [29, 30]. However, in patients with genotype 1a with Q80K polymorphism at baseline, the rates of SVR were reduced to 52% to 75%. In Japan, where genotype 1b is predominant and baseline Q80K is rarely observed, the phase III CONCERTO-1 trial showed that SMV-containing triple therapy increased the SVR rate to 89% in previously untreated patients [31]. In addition, the rate of SVR with triple therapy including SMV was 83% in previously untreated patients with genotype 4, in whom the baseline Q80K substitution is rarely detectable [32]. Although approved only in Japan, vaniprevir can produce similar SVR rates [33].

SOF is a nucleotide analog HCV NS5B polymerase inhibitor with pan-genotypic antiviral potency. In the phase III NEUTRINO trial, a 12-week regimen of SOF plus PEG-IFN and RBV resulted in SVR in 92% of previously untreated patients with genotype 1a, 82% of those with genotype 1b, and 96% of those with genotype 4 [34].

DCV is an NS5A replication complex inhibitor. The phase IIb COMMAND-1 study indicated that 60 mg of DCV plus PEG-IFN and RBV for 12 weeks followed by PEG-IFN and RBV with or without DCV for 12 weeks or PEG-IFN and RBV alone for 36 weeks according to protocol-defined response yielded SVR in 55% of previously untreated patients with genotype 1a, 77% of those with genotype 1b, and 100% of those with genotype 4 [35]. The rate of SVR to 60 mg of DCV-containing triple therapy was 90% to 100% in previously untreated Japanese patients with genotype 1b [36, 37].

However, the IFN-based regimens are no longer recommended in the AASLD and EASL guidelines, at least as first-line therapy for treatment-naïve patients, because they are inferior to IFN-free oral DAA combinations in terms of both their safety and tolerability profiles.

### 3.2. Genotypes 2/3

As described below, an IFN-free SOF and RBV combination is the current standard of care for patients infected with genotypes 2 and 3. However, previously treated patients (especially those with genotype 3) show suboptimal SVR rates. The phase IIb LONESTAR-2 study indicated that SVR was achieved in 96% of previously treated patients with genotype 2 and in 83% of those with genotype 3 by SOF plus PEG-IFN and RBV for 12 weeks [38].

### 3.3. Genotypes 5/6

In the phase III NEUTRINO trial of a 12-week regimen of SOF plus PEG-IFN and RBV, one patient with genotype 5 and all six patients with genotype 6 showed SVR [34]. However, evidence with which to recommend any regimen for patients with genotype 5 or 6 is still lacking.

## 4. IFN-Free DAA Therapy

### 4.1. Genotypes 1/4

An IFN-free combination of SOF and RBV is not recommended for patients with genotype 1 because the efficacy of the regimen was suboptimal in earlier arms of the phase II ELECTRON trial [39]. Some regimens involving combinations of other DAAs with SOF have since been tested in clinical trials.

The phase IIb COSMOS study showed that the NS5B inhibitor SOF and NS3/4A inhibitor SMV with or without RBV for 12 to 24 weeks resulted in SVR in 90% of previously treated patients with mild fibrosis and 94% of previously treated or untreated patients with advanced fibrosis [40]. RBV, treatment duration, and SMV-resistant baseline Q80K polymorphism had little impact on SVR in this trial.

The phase II AI444040 study indicated that the NS5B inhibitor SOF and NS5A inhibitor DCV with or without RBV for 12 to 24 weeks produced SVR in 98% of previously untreated patients with genotype 1 [41]. An SVR was also obtained with this regimen for 24 weeks in 98% of patients who had previous virological failure with NS3/4A inhibitor TVR or BOC.

In the phase III ION-1 trial, 97% to 99% of previously untreated patients with genotype 1 achieved SVR with a once-daily, fixed-dose combination of the NS5B inhibitor SOF and NS5A inhibitor LDV with or without RBV for 12 to 24 weeks, regardless of the addition of RBV or treatment duration [42]. The results of the phase III ION-3 trial suggested that the duration of treatment with SOF and LDV may be shortened to 8 weeks in treatment-naïve patients with genotype 1 without cirrhosis [43]. The phase III ION-2 trial showed that among patients with genotype 1 previously treated with PEG-IFN and RBV with or without NS3/4A inhibitor the SVR rates with a combination of SOF and LDV with or without RBV were 94% to 96% when given for 12 weeks and 99% when given for 24 weeks [44]. In the Japanese phase III trial, SOF and LDV with or without RBV for 12 weeks yielded SVR in 98% of treatment-naïve patients and 100% of previously treated patients with genotype 1 [45]. The fixed-dose SOF and LDV combination with or without RBV was also efficacious in cirrhotic patients with genotype 1 that had been unresponsive to previous NS3/4A protease inhibitor therapy in the phase II SIRIUS trial [46]. A proof-of-concept phase IIa study suggested that addition of a third potent DAA, such as the NS3/4A inhibitor GS-9451 or the nonnucleoside NS5B inhibitor GS-9669, to this fixed-dose combination can shorten the treatment duration to 6 weeks in noncirrhotic patients with genotype 1 [47]. The SOF and LDV combination appears to be effective for patients with genotype 4 [48]. ASTRAL, clinical trials of SOF plus the next-generation NS5A inhibitor, velpatasvir (GS-5816), have just been published [49]. In detail, SOF and velpatasvir combination therapy for 12 weeks produced SVR in 98% and 100% of patients with genotype 1 and genotype 4, respectively.

In the preliminary AI447-011 study to assess the efficacy of the IFN-free combination of the NS5A inhibitor DCV and the NS3/4A inhibitor ASV, both patients with genotype 1b achieved SVR, compared with only two of nine (22%) patients with genotype 1a [50]. The Japanese phase III AI447-026 study of a 24-week DCV and ASV combination thus included only patients with genotype 1b; 89% of patients who were intolerant to or ineligible for IFN and 80% of patients with a null response to IFN achieved SVR [51]. The regimen has already been approved in Japan, and the results of the Japanese study were reproduced in the multinational phase III multicohort HALLMARK-DUAL trial [52]. However, the efficacy of the DCV and ASV regimen is markedly affected by baseline NS5A RAV. In this study, baseline L31M/V substitutions were detected in 5% of patients, only 41% of whom achieved SVR, and baseline Y93H substitutions were detected in 8% of patients, only 38% of whom achieved SVR. In the phase III UNITY-1 study, addition of the nonnucleoside NS5B inhibitor beclabuvir to DCV and ASV for 12 weeks provided SVR in 92% and 89% of treatment-naïve and previously treated noncirrhotic patients with genotype 1, respectively, irrespective of baseline NS5A RAV [53].

In the phase III SAPPHIRE-I study, among previously untreated patients without cirrhosis, the NS3/4A inhibitor paritaprevir boosted with ritonavir, the NS5A inhibitor ombitasvir, and the nonnucleoside NS5B inhibitor dasabuvir with RBV for 12 weeks produced SVR in 95% and 98% of patients with genotype 1a and genotype 1b, respectively [54]. In the phase III PEARL-IV study, among previously untreated patients with genotype 1a without cirrhosis who were treated with the all-oral three-DAA regimen for 12 weeks, the rate of SVR was 97% with RBV and 90% without RBV, suggesting that RBV is necessary for patients with genotype 1a [55]. In the phase III TURQUOISE-II study, among previously treated or untreated patients with genotype 1a with compensated cirrhosis who were treated with the all-oral three-DAA regimen with RBV, the rate of SVR was 89% in the 12-week arm and 95% in the 24-week arm, suggesting that a 24-week treatment period is preferable for cirrhotic patients [56]. In the phase IIb PEARL-I study, a two-DAA regimen consisting of paritaprevir (with ritonavir) and ombitasvir with or without RBV was sufficient for treatment-naïve or previously treated patients with genotype 4 without cirrhosis [57]. The Japanese phase IIb study suggested that the two-DAA regimen without RBV was effective for previously treated patients with genotype 1b without cirrhosis [58].

The phase III C-EDGE treatment-naive study showed that among cirrhotic and noncirrhotic treatment-naïve patients once-daily fixed-dose treatment with a combination of the second-generation NS3/4A inhibitor grazoprevir and NS5A inhibitor elbasvir for 12 weeks resulted in SVR in 95% of those with genotype 1a and 99% of those with genotype 1b [59].

### 4.2. Genotypes 2/3

Among previously untreated patients with or without cirrhosis who received SOF and RBV for 12 weeks in the phase III FISSION study, the rate of SVR was 97% in those with genotype 2 and 56% in those with genotype 3 [34]. In the phase III FUSION study of SOF and RBV in previously treated patients with or without cirrhosis, the rates of SVR in genotype 2 were 86% in the 12-week group and 94% in the 16-week group, and the corresponding rates of SVR in genotype 3 were 30% and 62%, respectively [60]. These results suggested that extended treatment is beneficial for previously treated patients, but a 16-week treatment period is still insufficient for patients with genotype 3. Among previously treated or untreated patients who received SOF and RBV in the phase III VALENCE study, SVR was achieved in 93% of those with genotype 2 who were treated for 12 weeks and 85% of those with genotype 3 who were treated for 24 weeks (91% and 68% of those without and with cirrhosis, resp.) [61]. In a Japanese phase III trial, SOF and RBV for 12 weeks resulted in SVR in 98% of treatment-naïve and 95% of previously treated patients with genotype 2 [62].

The phase III ALLY-3 study to evaluate the 12-week regimen of SOF plus DCV in treatment-naïve and previously treated patients with genotype 3 showed SVR rates of 96% in patients without cirrhosis and 63% in those with cirrhosis [63]. Additional evaluation to optimize efficacy in patients with genotype 3 with cirrhosis is currently underway.

### 4.3. Genotypes 5/6

Little information is available regarding the efficacy of IFN-free DAA regimens, particularly in patients with genotype 5. In a preliminary report, 96% of treatment-naïve and previously treated patients with genotype 6 showed SVR with a 12-week combination of SOF and LDV [64].

## 5. Appearance of HCV in Patients Who Previously Relapsed or Showed Breakthrough after DAA Treatment

Patients treated with an NS3 protease inhibitor, an NS5A inhibitor, or a nonnucleoside inhibitor of NS5B who failed to achieve SVR were found to have viruses with amino acid substitutions that confer drug resistance in the NS3 protease, NS5A, and NS5B regions, respectively. For example, resistance-associated variants (RAVs) with NS3 positions V36A/G/I/L/M, T54A/S, I132V (genotype 1a only), R155G/K/T/M, A156F/N/S/T/V, and D168N were identified after failure of treatment with TVR and combined PEG-IFN/RBV therapy [65]. In addition, for patients in whom SMV/PEG-IFN/RBV therapy failed, RAVs were identified with NS3 positions 80, 122, 155, and 168 (mainly R155K in genotype 1a with and without Q80K, and D168V in genotype 1b) at the time of failure [66]. RAVs with both NS5A and NS3 amino acid substitutions emerged in patients in whom IFN-free DAA therapy with DCV and ASV failed. Of note, NS5A variants with both L31V/M and Y93C/N show strong drug resistance [67, 68]. In comparison, few reports have described the detection of NS5B-polymerase-inhibitor-resistant HCV (S282T in NS5B) after failure of SOF combined antiviral therapy. Currently, SOF has the highest barrier to drug resistance.

Another interesting feature of RAVs after DAA failure is the change in the prevalence of RAVs over time. NS5A resistance variants (Q30E/R, L31V/M, and Y93C/N) persisted, while NS3 resistance variants (V36M, R155K, and D168A/E/V/Y) generally decreased [65, 68].

## 6. Strategy of DAA Retreatment for Patients with Previous DAA Therapy

Although DAA combination therapy can achieve a high SVR rate (>90%), approximately 5% of patients treated with DAAs will fall into relapse or viral breakthrough. It is necessary to consider retreatment for patients with failure of DAA therapy. It was speculated that previously used DAAs (with the same resistance profile) may lose their anti-HCV effect in patients following DAA failure.* In vitro* analysis of combinations with DCV, ASV plus beclabuvir, and DCV, ASV, and beclabuvir plus SOF efficiently cleared resistant replicons to both DCV and ASV. On the other hand, SOF plus SMV and SOF plus LDV did not inhibit the growth of DCV- plus ASV-resistant replicons [69]. Another unique trial has just been reported. It showed that an NS5A inhibitor analogue (Syn-395) induced conformational change in the resistant NS5A protein and that RAVs became resensitized to DCV in* in vitro* and* in vivo* analyses [70].

The phase II C-SALVAGE study evaluated a combination of grazoprevir (NS3/4A protease inhibitor) and elbasvir (NS5A inhibitor) with RBV for patients with chronic HCV genotype 1 infection in whom licensed DAA-containing therapy had failed. The patients had failed to achieve SVR by PEG-IFN and RBV plus either BOC, TVR, or SMV. Grazoprevir (100 mg)/elbasvir (50 mg) QD with weight-based RBV BID for 12 weeks achieved a 96.2% (76/79) overall SVR12 rate, including 43 of 43 (100.0%) patients without baseline RAV, 31 of 34 (91.2%) patients with baseline NS3 RAV, 6 of 8 (75.0%) patients with baseline NS5A RAV, and 4 of 6 (66.7%) patients with both baseline NS3 and RAV [71]. In a phase IIa open-label study, 14 patients with HCV GT-1 who relapsed after treatment with SOF plus RBV for 24 weeks were retreated with SOF plus LDV for 12 weeks. All 14 patients achieved SVR, including one patient with NS5B S282T mutation after previous SOF plus RBV therapy [72]. Taken together, it is speculated that retreatment with DAAs of the same class plus additional DAAs with a different mechanism of action and/or new DAAs could achieve SVR in patients that had previously fallen into relapse or breakthrough after DAA treatment.

## 7. Prognosis after Achieving SVR by DAAs

IFN-based therapy is well known to prevent the development of hepatocellular carcinoma (HCC) if HCV can be completely eradicated from patients with CHC. In addition, IFN-based therapy could improve the hepatic fibrosis stage in patients who achieved SVR [73–75]. In general, overall survival is prolonged in patients who achieve SVR. On the other hand, patients with advanced hepatic fibrosis or cirrhosis continue to be at risk for the development of HCC after eradication of HCV. The cumulative rate of HCC development is reportedly 1% to 3% in SVR patients in Japan [76–78]. Elderly patients and those with advanced fibrosis prior to IFN-based treatment are at risk for the development of HCC after achieving SVR [79]. Compared with IFN-based therapy, DAAs may be more suitable for elderly patients and patients with hepatic cirrhosis. In addition, IFN-free DAA therapy achieved a higher SVR rate. However, which DAA therapy will reduce the incidence of HCC is questionable. It is necessary to evaluate the long-term prognosis of patients treated with DAAs.

In addition to eradication of HCV, DAA therapy can improve lipid metabolism in patients with SVR. That is, during SOF and RBV therapy for patients with HCV genotype 1, serum lipid concentrations were altered and intrahepatic expression of fatty acid metabolism and lipid transport genes were also changed [80]. With regard to other outcomes in patients treated with ledipasvir and SOF combination therapy, four questionnaires—CLDQ-HCV, SF36, FACIT-F, and WPAI:SHR—were used to evaluate health-related quality of life (HRQL) and work productivity in patients treated with IFN-free therapy. The results indicated that viral eradication with IFN-free therapy led to improvement in HRQL regardless of the hepatic fibrosis stage at baseline [81].

## 8. Conclusions

DAAs can eradicate HCV in almost all patients with chronic liver disease, including advanced fibrosis, and can inhibit continuous inflammation in the liver. However, this is the treatment goal for HCV infection, but not for liver disease. It has not been confirmed that DAA can improve hepatic fibrosis and prohibit hepatocarcinogenesis. Therefore, it is necessary to evaluate patients' hepatic pathogenesis after achievement of SVR by DAA therapy.

Another problem associated with DAA treatment is that compared with interferon-based therapy new DAA regimens are too expensive to be accessible to all patients with HCV. Therefore, it is necessary to consider not only efficacy, but also cost in selection of anti-HCV therapy.

## Figures and Tables

**Table 1:** Profile of NS3/4A protease inhibitors.

Generation			Active against HCV genotype	Genetic barriers	Resistant association variants					
1st, 1st-wave	VX-950	Telaprevir	1a/1b, 2	Low	V36A/M		R155K/T/Q	A156S/D/T/V		
1st, 1st-wave		Boceprevir	1a/1b, 2	Low	V36A/M		R155K/T/Q	A156S/D/T/V		
1st, 2nd-wave	BI 201335	Faldaprevir	1a/1b, 2	Moderate			R155K/T/Q		D168A/V/T/H	V170A/T
1st, 2nd-wave	TMC-435	Simeprevir	1, 2, and 4–6	Moderate		Q80R/K			D168A/V/T/H	
1st, 2nd-wave	MK-7009	Vaniprevir	1a/1b	Moderate			R155K/T/Q	A156S/D/T/V	D168A/V/T/H	
1st, 2nd-wave	BMS-650032	Asunaprevir	1, 4	Moderate		Q80R/K	R155K/T/Q		D168A/V/T/H	
1st, 2nd-wave	ABT-450	Paritaprevir	1	Moderate			R155K/T/Q		D168A/V/T/H	
2nd	GS-9857		1–4				R155K/T/Q	A156S/D/T/V	D168A/V/T/H	
2nd	ACH-1625	Sovaprevir								
2nd	MK-5172	Grazoprevir	1a/1b, 2, and 4–6	High			R155K/T/Q	A156S/D/T/V	D168A/V/T/H	

Reference [16] Vermehren and Sarrazin 2012.

**Table 2 tab2:** Profile of NS5A inhibitors.

Generation			Active against HCV genotype	Genetic barriers	Resistant association variants			
1st	BMS-790052	Daclatasvir	1b > 2a > 1a	Moderate			L31F/M/V	Y93C/H/N
1st	GS-5885	Ledipasvir	1a, 1b	Moderate			L31F/M/V	Y93C/H/N
1st	ABT-267	Ombitasvir	1 > 2–6	Moderate	M28T	Q30E/R		Y93C/H/N

Broad activity	MK-8742	Elbasvir	1–4	Unavailable	M28T	Q30L/R	L31<	Y93H/N
2nd	GS-5816	Velpatasvir	1–6	Unavailable				
2nd	ACH-3102		1–5	High				Y93H

Reference [23] Kohler et al. 2014.

**Table 3:** Profile of NS5B inhibitors.

		Binding site	Active against HCV genotype	Genetic barriers	Resistant association variants						
Nucleotide											
GS-7977	Sofosbuvir		1a, 1b, and 2–6	High	S282T						

Nonnucleoside											
BMS-791325	Beclabuvir	Site I		Moderate	A421V	P495S/Q/L/A/T					
ABT-333	Dasabuvir	Site III		Moderate	C316Y/N	S368T	M414T/I/V/L	Y448C/H	G554D/S	S556G	D559G
GS9669		Site II		Moderate	L419S	R422K	M423T/I/V/T	I482L/V/T	A486/V/I/T/M	V494A	
MK-3682				Moderate							

Reference [16] Vermehren and Sarrazin 2012.
